# State Control of Public Health

**Published:** 1899-03

**Authors:** 


### State Control of Public Health.

There can be no argument of weight offered against the State assuming the responsibility of caring for the health of its citizens and making such laws as will best protect the general public against disease.

The statute books of the Commonwealth have spread upon their pages enactments to protect its citizens from violence, to secure to them the quiet enjoyment of their possessions, to enable them to live

*A Treatise on Fractures and Dislocations, by Lewis A. Stimson, B. A., M. D., Professor of Surgery in Cornell University Medical College, New York. Lea Brothers & Co. Just issued.

in liberty and at peace; to advance their material and spiritual welfare. But these laws are far from perfect, hence the attention of a large body of legislators is necessary, from time to time, to improve them, and by these improvements make them apply to the ever changing environment of our progressive civilization.

Education is everywhere recognized as the force by which our modern world has reached its present elevated plane. The country's law-givers and wise men, alive to this great fact, have done everything in their power to encourage education. Public schools have been established, literary colleges endorsed, and non-attendance at school has been legislated into a misdemeanor. These educational institutions are under the supervision of the State.

Medical schools do not come under this head. It is true in a few States medical education is under State supervision, and in many it is controlled by examining boards, while in some it is hampered by adverse legal enactments.

The mastery of both surgery and medicine depends upon an accurate knowledge of human anatomy, which can only be obtained through dissection of the human body.

Whenever a State legislature makes is a felony to rob a grave, without providing a legal way by which dissecting material may be procured, then and at that time the State deals a serious blow to medical education, and indirectly to the physical well being of her citizens.

Sound medical education is of more value to a community than is any health board, and God save us from a health board that has not at its head a thoroughly educated physician. If it is the duty of the State to protect her citizens against the assaults of an enemy, the inroads of smallpox or yellow fever, it is equally her duty to protect them, when sick, from the dire results of fraud or ignorance at the hands of legalized practitioners of medicine.

Lawmaking in regard to anything so sacred as human life is a serious business to a man who possesses a conscience. The sin of omission is little less than that of commission, and the legislator who helps to enact a law by which an uneducated man is permitted to go forth, and in his conceited ignorance do unto death a sick, unsuspecting victim, is equally guilty with him who commits the deed. All that prevents this crime from being classed with murder is the lack of intention. Ignorance is not a valid excuse before the law, yet legislators make it so when by these enactments it is possible for an unqualified man to hold a position in which he gambles with human life. Strange to say, politicians do not think of the responsibilities when they appoint such an one to the charge of these sacred trusts. What pangs they will suffer when their consciences are called upon for their balance sheets!

In the interests of the citizens medical education should not only be fostered, but it should be regulated by the State. Diploma mills have existed and do now exist in this country, to the great injury of sound medical teaching. Supervision and careful inspection is healthy in both public and private institutions. It never does harm.

The public welfare demands educated doctors, and it is clearly the State's business to know beyond a doubt that the holder of a license to practice medicine is qualified to do so. The system of examining applicants by a board of regents, as practiced in many States, is efficient. The examining board in our own State of Tennessee has done good service since its establishment, and we trust it may be long continued, to do such work in the future.

In the interests of medical education, our legislators should make such provisions as would place at the disposal of the various medical colleges in the State, for the purposes of dissection, the bodies of all friendless paupers who may die in the State institutions for charity.

Our readers are asked to consider this subject carefully and use their personal influence with their representatives at the State Capitol to secure these necessary enactments.—*Medical and Surgical Bulletin, Nashville.*

